# Flow-reactor synthesis of octahedral Pt–Ni nanoparticles modified with Au and their evaluation as oxygen reduction reaction catalysts

**DOI:** 10.1039/d5na01009h

**Published:** 2025-12-02

**Authors:** Tomoyuki Nagai, Akira Kuwaki, Kensaku Kodama

**Affiliations:** a Toyota Central R&D Labs. Inc. 41-1 Yokomichi Nagakute Aichi 480-1192 Japan ngtmyk@mosk.tytlabs.co.jp

## Abstract

Developing oxygen reduction reaction (ORR) catalysts with both high catalytic activity and durability is essential for the commercialization of polymer electrolyte fuel cells (PEFCs). One promising strategy to simultaneously enhance the catalytic activity and durability of Pt-based catalysts is surface modification with Au, which has led to substantial durability improvements in various catalysts such as bulk electrodes, nanowires, and core–shell structures. In this study, octahedral Pt–Ni nanoparticles (oct-Pt–Ni NPs), which are known to exhibit exceptionally high ORR activity, were synthesized and modified with Au using a continuous flow reactor to investigate the effects of Au modification on catalytic activity and durability. The flow synthesis enabled uniform Au deposition on individual nanoparticles owing to the rapid mixing and homogeneous contact between the Au precursor and Pt–Ni nanoparticles. Electrochemical measurements revealed that Au modification enhanced the specific activity (SA) by up to 1.5 times, while the mass activity (MA) remained nearly unchanged owing to the decrease in electrochemical surface area of Pt. The activity enhancement suggests that Au atoms promote the catalytic activity of the neighboring Au-free Pt–Ni sites as previously reported. In contrast, the MA of Au-modified oct-Pt–Ni NPs rapidly decreased within several hundred potential cycles along with the decrease in the SA, indicating that Au atoms on the Pt–Ni nanoparticles could not effectively suppress Ni leaching or morphological transformation. These results suggest that the beneficial effect of Au modification on durability is limited for shape-sensitive catalysts with numerous vulnerable edges and corners such as oct-Pt–Ni nanoparticles, unlike the spherical or nanowire catalysts with smooth or well-faceted surfaces.

## Abstract 2

Au was uniformly deposited on octahedral Pt–Ni nanoparticles *via* a continuous-flow reactor. Although Au enhances ORR activity, it provides limited suppression of Ni leaching and structural degradation.

## Graphical abstract

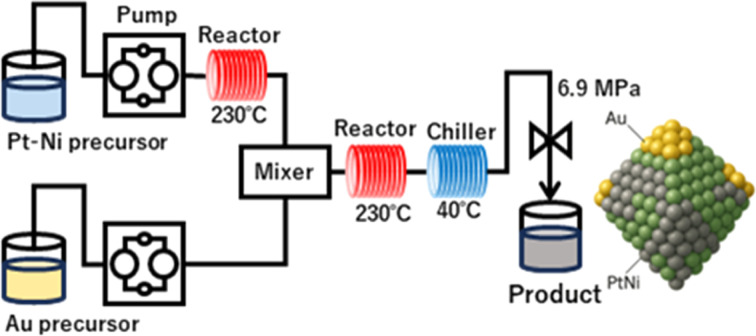

## Introduction

Achieving both high catalytic activity and durability in oxygen reduction reaction (ORR) catalysts is crucial for the practical application of polymer electrolyte fuel cells (PEFCs). High catalytic activity improves fuel economy by reducing the reaction overpotential and the associated heat loss, thereby enhancing the overall fuel efficiency. Alternatively, it minimizes the required amount of precious metals, which reduces catalyst cost and conserves critical resources. High durability, on the other hand, ensures reliable long-term operation and avoids the high cost associated with replacing the PEFC stack.

A promising strategy to simultaneously enhance the catalytic activity and durability is surface modification of Pt-based catalysts with foreign elements.^1–4^ Among such approaches, Au modification of Pt-based catalysts has attracted considerable attention. Au modification typically reduces the electrochemical surface area of Pt (ECSA) by partially covering the active Pt surface, yet it has been shown to increase the specific activity (SA) of the remaining Au-free Pt sites. Furthermore, when Au atoms are selectively deposited on vulnerable, low-coordinated Pt sites, the mass activity (MA) can also be enhanced despite the reduced ECSA. These effects were first reported for pure Pt nanoparticles by A. Zhang *et al.*,^5^ subsequently supported through theoretical calculations and model electrode experiments,^6–8^ and later confirmed for other Pt-based catalysts as well.^9–17^ For instance, Au-modified Pt–Ni nanowires, one of the so-called shape-controlled Pt catalysts,^18–23^ exhibited exceptionally high MA exceeding 3000 A g^−1^ together with remarkable durability.^14^

Another representative high-activity Pt-based catalyst is the octahedral Pt–Ni nanoparticles (oct-Pt–Ni NPs). However, it suffers from poor stability, which mainly originates from the rapid dissolution of low-coordinated Pt atoms located at the edges and corners of the nanoparticles, leading to the loss of the octahedral morphology and the leaching of internal Ni atoms.^24^ Therefore, protecting these vulnerable sites with more stable Au atoms is expected to improve the overall stability of the octa-Pt–Ni NPs. In this study, we synthesized Au-modified oct-Pt–Ni nanoparticles (Au-oct-Pt–Ni NPs) and investigated the influence of Au modification on their catalytic activity and durability through rotating disk electrode (RDE) measurements and characterization techniques. Conventionally, Au modification is conducted *via* a two-step process involving Cu underpotential deposition (Cu-UPD), followed by galvanic replacement with Au ions. However, the galvanic replacement proceeds very rapidly owing to the large potential difference between Cu dissolution and Au deposition, and can also occur at remote sites through electronic conduction within catalyst aggregates. As a result, Au tends to deposit locally at easily accessible surface regions of these aggregates (see SI). To achieve more uniform Au deposition on oct-Pt–Ni NPs, we employed a continuous flow reactor that enables rapid mixing and homogeneous contact between the Au precursor and Pt–Ni nanoparticles. This approach not only provides well-defined catalyst surfaces for evaluating the effects of Au modification on activity and durability but also offers inherent scalability for practical catalyst synthesis.

## Experimental

### Chemicals

Platinum(ii) acetylacetonate (Pt(acac)_2_, 97%), nickel(ii) acetylacetonate (Ni(acac)_2_, 95%), tungsten hexacarbonyl (W(CO)_6_, 97%), oleylamine (OAm, 70%), oleic acid (OAc, 90%), and tetraoctylammonium bromide (TOAB, 98%) were obtained from Sigma-Aldrich. Hydrogen tetrachloroaurate(iii) tetrahydrate (HAuCl_4_·4H_2_O, 99.0%), toluene (99.5%), ethanol (99.5%), and 2-propanol (99.5%) were obtained from FUJIFILM Wako Pure Chemical Corporation. Perchloric acid (Ultrapur) was obtained from Kanto Chemical Co., Inc. All chemicals were used as received.

### Preparation of the Pt–Ni precursor solution

97.3 mg of Pt(acac)_2_, 62.9 mg of Ni(acac)_2_, and 148.2 mg of W(CO)_6_ were dissolved with 3.29 mL of OAm and 0.283 mL of OAc in toluene, and the total volume was adjusted to 40 mL so that the concentrations of Pt(acac)_2_, Ni(acac)_2_, W(CO)_6_, OAm, and OAc were 6 mM, 6 mM, 0.01 M, 0.2 M, and 0.02 M, respectively.

### Preparation of the Au precursor solution

33.3 mg of HAuCl_4_·4H_2_O was dissolved in 4 mL of water to prepare a 20 mM aqueous Au solution. Separately, 223 mg of TOAB was dissolved in 4 mL of toluene to obtain 0.1 M TOAB solution. Then, the 20 mM Au solution was vigorously mixed with the 0.1 M TOAB toluene solution to transfer the AuCl_4_^−^ ions into the toluene phase *via* phase transfer catalysis.^25^ The aqueous phase was then removed, and residual water in the toluene was eliminated using molecular sieves. Subsequently, 2.09 mL, 1.00 mL, and 0.49 mL of the resulting Au-toluene solution (approximately 20 mM) were each diluted to 40 mL to prepare 1.04 mM, 0.5 mM, and 0.245 mM Au precursor solutions, corresponding to Au/(Au + Pt + Ni) atomic ratios of 8%, 4%, and 2%, respectively.

### Synthesis of Au-modified oct-Pt–Ni nanoparticles

**Fig. 1:**
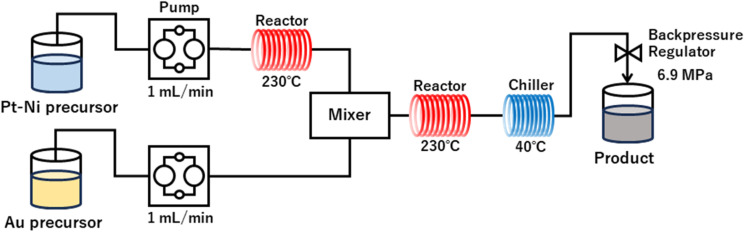
Continuous flow reactor configuration for Au-oct-Pt–Ni NPs synthesis.

Au-oct-Pt–Ni NPs were synthesized using a continuous-flow reactor, following a procedure modified from our previous report.^24^ A schematic diagram of the continuous-flow reactor is shown in Fig. 1. The setup consists of two dual-plunger pumps (YMCK-12-13-P, YMC), a back-pressure regulator (U-469 with P-796, IDEX Health & Science LLC.), a T-shaped stainless-steel mixer (SS-100-3, Swagelok), two hand-made tubular reactors, and a stainless-steel heat-exchanger connected to a low-temperature thermostat (E200, LAUDA). Each reactor was constructed from 1/16-inch stainless-steel tubing (inner diameter: 1.0 mm) coiled around an aluminium block equipped with a cartridge heater and a thermocouple (MISUMI).

The Pt–Ni precursor solution was pumped at a flow rate of 1 mL min^−1^ through the first reactor at 230 °C (length: 10 m). The residence time in the reactor was approximately 8 min, during which oct-Pt–Ni NPs were formed. The resulting suspension was then mixed with the Au precursor solution injected at a flow rate of 1 mL min^−1^, and subsequently passed through the second reactor at 230 °C (length: 20 m) at a combined flow rate of 2 mL min^−1^. The residence time in the second reactor was also approximately 8 min for Au deposition on oct-Pt–Ni NPs. The reactor outlet was cooled to 40 °C using a water chiller, and the internal pressure was maintained at 6.9 MPa with a back-pressure regulator to prevent solvent boiling.

### Loading nanoparticles onto carbon

The synthesized nanoparticles were loaded onto carbon as follows. The Au-oct-Pt–Ni NPs dispersed in 70 mL of the product solution were precipitated by adding 2-propanol, followed by centrifugation at 6000×*g* for 5 min. The supernatant was removed, and the precipitate was redispersed in 70 mL of hexane, containing approximately 41 mg of Pt estimated from the precursor amounts. The resulting dispersion was mixed with 70 mL of a hexane dispersion containing about 100 mg of carbon black (Vulcan XC-72R) and sonicated in an ice bath for 60 min to deposit nanoparticles onto the carbon support. The obtained Au-oct-Pt–Ni/C catalyst was washed more than five times with hexane through repeated centrifugation and redispersion. Finally, the catalyst was dispersed in ethanol, collected by vacuum filtration, and dried under vacuum with silica-gel at room temperature for more than 4 h. A reference catalyst without Au modification (0%Au-oct-Pt–Ni/C) was prepared under identical conditions, except that pure toluene was used instead of the Au precursor solution.

In preparing the carbon-supported catalyst, the amount of carbon black was set so that the Pt loading of the catalyst would be 25 wt%, assuming that all the Pt, Ni, and Au precursors were completely converted into nanoparticles and that all the Au-oct-Pt–Ni NPs would be loaded on carbon in the loading process. In practice, however, as shown later, the measured Pt loadings were approximately 20 wt%, which was slightly lower than the designed value due to material losses during synthesis and washing processes.

### Determination of Pt loading in the catalysts

Approximately 4 mg of Au-oct-Pt–Ni/C catalyst was calcined in air at 800 °C for 30 min. The residue was dissolved in aqua regia at 90 °C and evaporated at 150 °C. A few drops of concentrated HCl were then added and evaporated again. The resulting metal chlorides were dissolved in 10 mL of ultrapure water. The Pt concentration was determined by absorption spectroscopy using the internal standard addition method with SnCl_2_ as a sensitizer, and the Pt loading of each catalyst was calculated accordingly. The detailed procedure for measuring the Pt concentration is described in the SI.

### Characterization of Au-oct-Pt–Ni/C

A field emission scanning transmission electron microscope (FE-STEM, JEM-ARM200F, JEOL) was used to observe the Au-oct-Pt–Ni/C catalysts. Compositional analysis was performed by energy dispersive X-ray spectroscopy (EDS) on more than 12 particles per sample. Crystal phases were examined using an X-ray diffractometer (XRD, Ultima IV, Rigaku Corporation).

### Electrochemical setup

Catalyst inks were prepared by mixing 5 mg of (Au-)oct-Pt–Ni/C with a 25 vol% 2-propanol aqueous solution and 5 wt% Nafion solution, followed by sonication in an ice bath for 60 min. A 10 µL of the ink was pipetted onto a polished glassy carbon (GC, 5 mm in diameter) and dried in air at 100 °C to prepare the catalyst-coated electrode. The Pt loading on the electrode was 7.5 µg-Pt cm^−2^, and the ionomer-to-carbon weight ratio (I/C) was 0.5. For comparison, an electrode using the commercial Pt/C catalyst (TEC10V30E, 29.1 wt% Pt, TKK) was also prepared under the same conditions, giving the same Pt loading on the electrode as that of the Au-oct-Pt–Ni/C catalysts.

All electrochemical measurements were conducted in 0.1 M HClO_4_ at room temperature using a three-electrode cell equipped with a rotating disk electrode (RDE). The catalyst-coated GC electrode was used as the working electrode (WE), while a platinized platinum electrode and a reversible hydrogen electrode (RHE) were used as the counter and reference electrodes, respectively.

### Catalyst conditioning

The catalysts on the WE were subjected to potential cycling between 0.05 and 1.05 V at a scan rate of 100 mV s^−1^ in an O_2_-saturated electrolyte at 400 rpm until the cathodic ORR current stabilized (typically ∼10 cycles).

### Electrochemical surface area (ECSA) measurement

Cyclic voltammograms (CVs) were recorded between 0.05 and 1.05 V at a scan rate of 50 mV s^−1^ in an Ar-saturated 0.1 M HClO_4_ electrolyte. The ECSA was calculated from the average charge of hydrogen adsorption and desorption, assuming 210 µC cm_Pt_^−2^, and normalized by the Pt amount on the WE. Although the hydrogen adsorption/desorption charge can vary with Pt–Ni composition,^26^ the relative comparison among the catalysts is valid because their Ni contents are similar (30–40%).^24^

### Activity measurement

Linear sweep voltammograms (LSVs) were recorded from 0.05 V to 1.05 V at a scan rate of 10 mV s^−1^ and a rotation speed of 1600 rpm in O_2_- and Ar-saturated 0.1 M HClO_4_ electrolytes. The solution resistance was measured at 10 kHz using an AC Milli-Ohm HITESTER (3560, HIOKI). The kinetic current (*i*_k_) was calculated using the mass-transfer correction:1
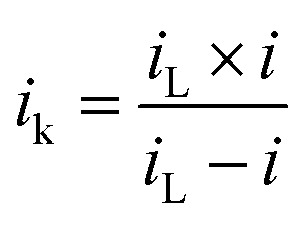
where *i* is the measured current, and *i*_L_ is the diffusion-limiting current. Background correction using the LSV in the Ar-saturated electrolyte and *iR* compensation were applied. The mass activity (MA) was determined by dividing *i*_k_ by the Pt amount on the WE, and the specific activity (SA) was obtained by dividing the MA by the ECSA.

### Accelerated durability test (ADT)

The accelerated durability (ADT) was carried out following the procedure described in our previous report.^27^ A square-wave potential cycling between 1.0 V (3 s) and 0.4 V (3 s) was applied in an O_2_-saturated 0.1 M HClO_4_ electrolyte at room temperature. The electrode rotation was stopped during potential cycles to minimize the dissolution of Pt ions from the catalyst layer into the bulk electrolyte.^28^ The ECSA, MA, and SA were measured after 0, 25, 50, 100, 200, 300, 400, 500, 1000, 1500, and 2000 cycles.

## Results and discussion

**Fig. 2:**
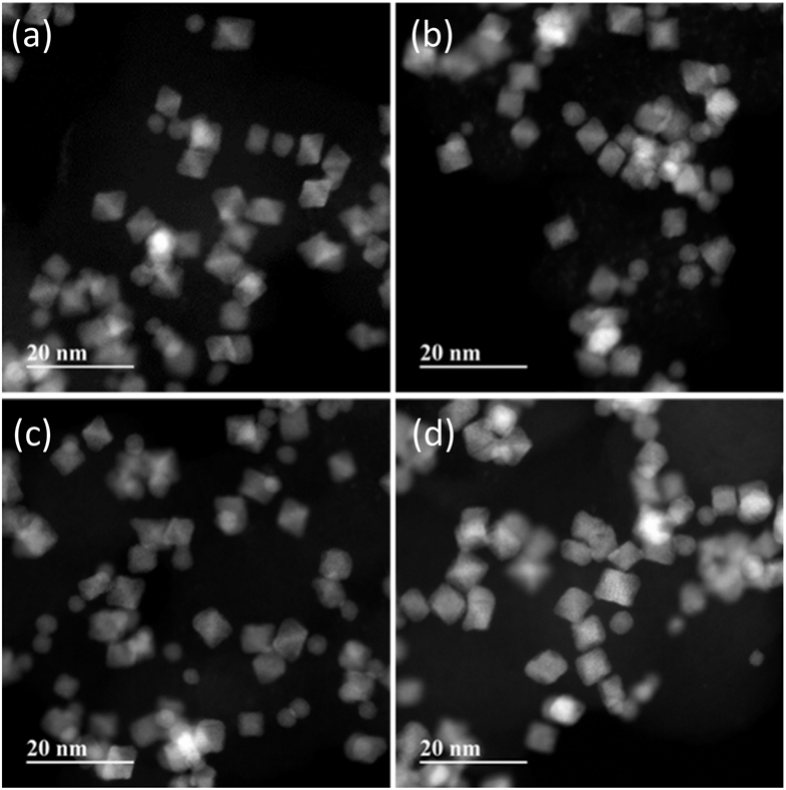
STEM images of Au-oct-Pt–Ni/C with different Au content: (a) 0% (unmodified), (b) 2%, (c) 4%, and (d) 8%.

**Fig. 3:**
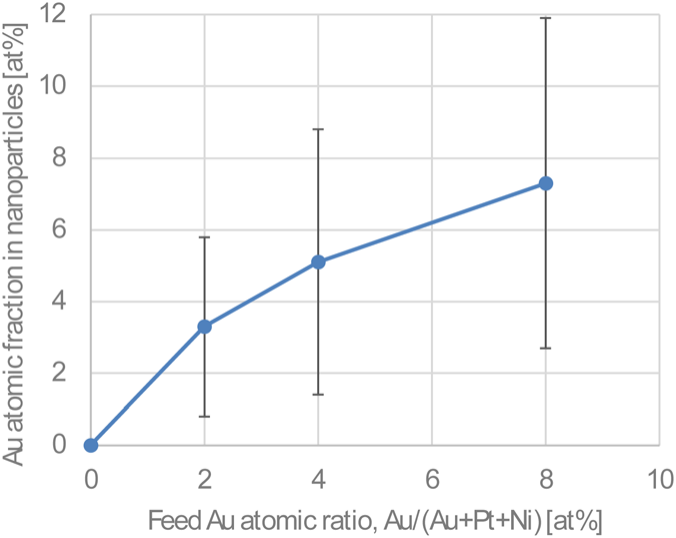
Correlation between the feed Au atomic ratio, Au/(Au + Pt + Ni) and the Au atomic fraction in nanoparticles estimated by EDS measurements. Error bars indicate standard deviations from more than 12 particles.

Fig. 2 shows STEM images of Au-oct-Pt–Ni/C prepared with different Au precursor concentrations. The Pt loadings of the catalysts were 19.0 wt%, 18.9 wt%, 20.7 wt%, and 21.7 wt%, respectively, with increasing Au contents (0%, 2%, 4%, 8%). Most of the nanoparticles exhibit well-defined octahedral shapes with an average size of approximately 6 nm, which remains unchanged regardless of the Au content. The corresponding particle-size distributions are provided in the SI (Fig. S3). The correlation between the Au atomic ratio in the feed solution and that in the resulting nanoparticles is shown in Fig. 3. The average Au content in the nanoparticles increases monotonically with the feed Au ratio. Although the EDS data show relatively large standard deviations due to the low Au signal at low Au contents and limited number of samples, the results clearly demonstrate that the Au composition can be effectively tuned by adjusting the Au concentration in the reactant solution. On the other hand, the Pt : Ni atomic ratio obtained from EDS (∼35%) was consistent with our previous report, where Pt and Ni contents were determined by absorption spectroscopy using SnCl_2_ and dimethylglyoxime as sensitizers, respectively.^24^

**Fig. 4:**
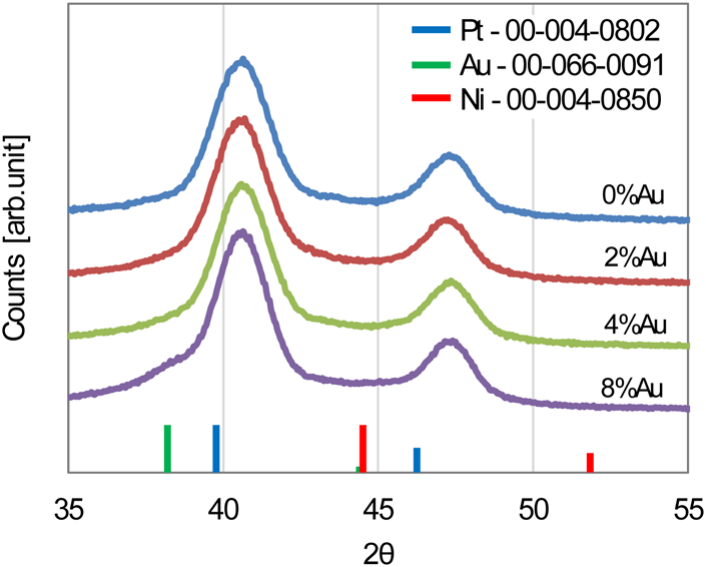
XRD patterns for Au-oct-Pt–Ni/C with different Au content. Vertical lines indicate the peak positions of Pt (blue), Ni (red) and Au (green) from the PDF database (ICDD).

Fig. 4 shows the XRD patterns of Au-oct-Pt–Ni/C with different Au contents. The diffraction peaks at approximately 41° and 47°, corresponding to the (111) and (200) planes of the face-centered cubic (fcc) structure of Pt, shift to higher angles compared with those of pure Pt, indicating the formation of a Pt–Ni solid solution.^24^ In contrast, the peak positions remain almost unchanged after Au modification, suggesting that Au does not incorporate into the Pt–Ni lattice. No distinct diffraction peaks of metallic Au are observed, except that the 8%Au–Pt–Ni/C exhibits a weak shoulder near 38°, assigned to Au(111) plane.

**Fig. 5:**
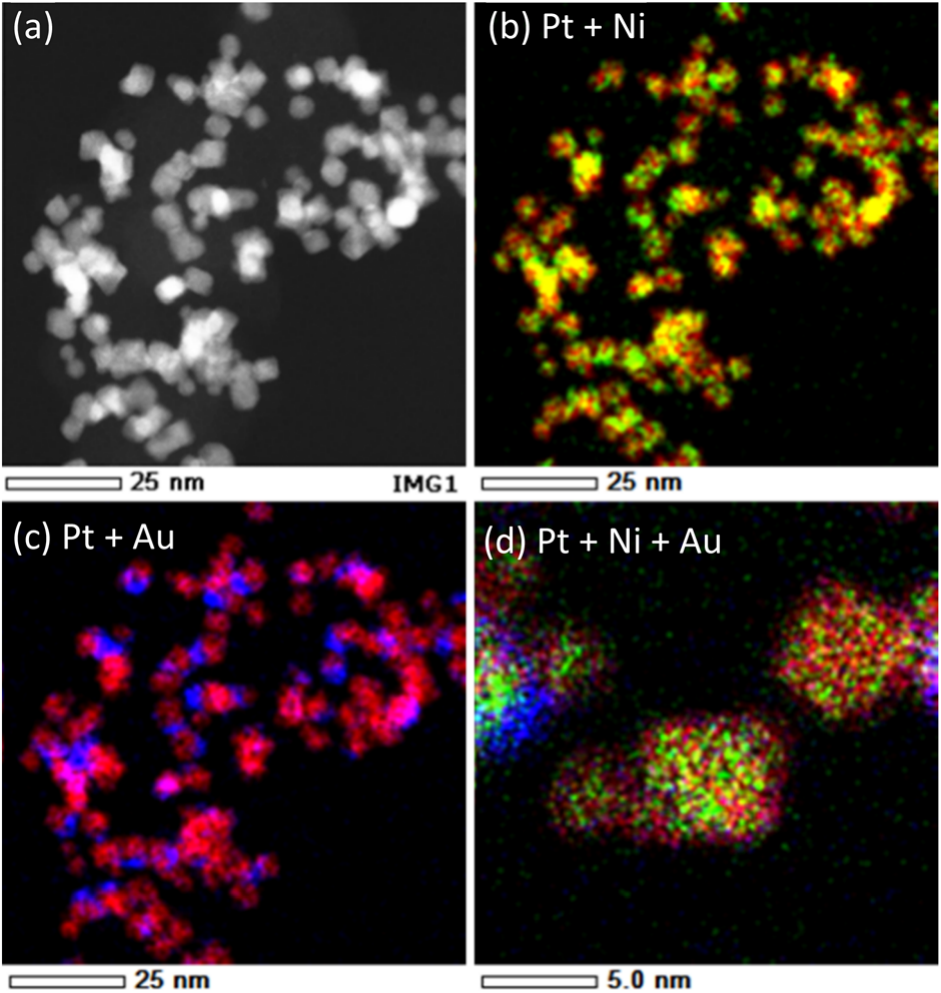
EDS elemental maps of 8%Au-oct-Pt–Ni/C (a) STEM image; (b) overlay of Pt (red) and Ni (green); (c) overlay of Pt (red) and Au (blue); (d) overly of Pt, Ni, and Au at high magnification.

The EDS elemental maps of 8%Au-oct-Pt–Ni/C (Fig. 5) further clarify the compositional arrangement of the nanoparticles. Pt and Ni are homogeneously distributed throughout the particles, confirming that the oct-Pt–Ni nanoparticles form a uniform alloy rather than a phase-separated or core–shell structure. In contrast, Au is broadly distributed across the catalyst and partially covers each Pt–Ni nanoparticle. Although the spatial resolution of EDS is insufficient to determine the detailed surface configuration of Au, such as whether it forms a monolayer or sub-monolayer, or whether it preferentially deposits at edges or corners, the combined EDS and XRD results indicate that Au resides primarily on the nanoparticle surfaces. No severe aggregation was observed even at 8% Au, where a weak shoulder attributed to Au(111) appears in the XRD pattern; however, excessive Au deposition at higher Au contents may lead to localized clustering on some particles.

**Fig. 6:**
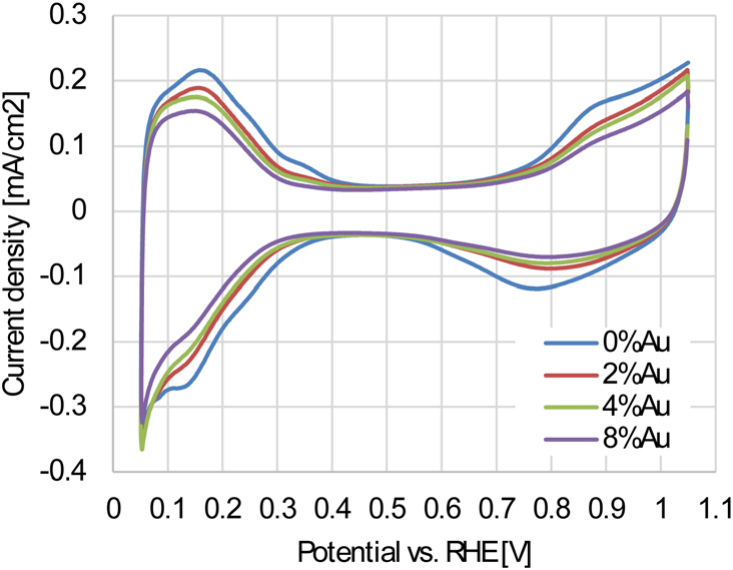
Cyclic voltammograms of the Au-oct-Pt–Ni/C with different Au content. The electrolyte is Ar-saturated 0.1 M HClO_4_, and the scan rate is 50 mV s^−1^.

**Fig. 7:**
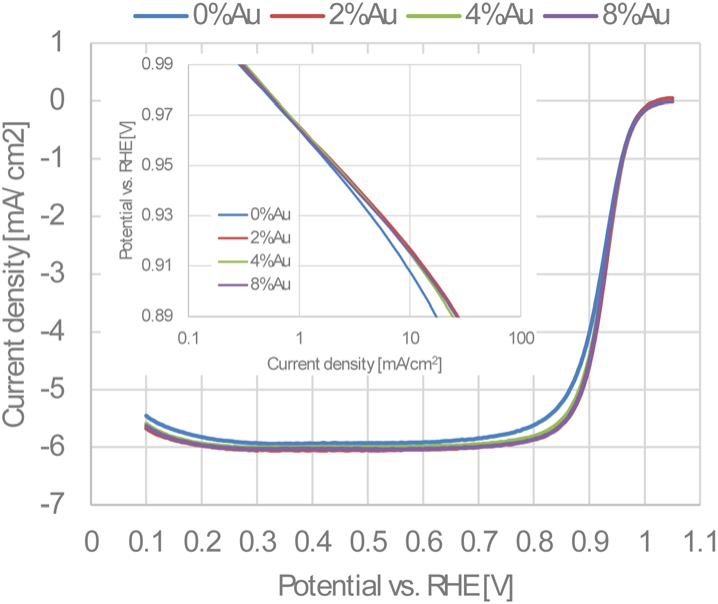
Linear sweep voltammograms of the Au-oct-Pt–Ni/C with different Au content. The electrolyte is O_2_-saturated 0.1 M HClO_4_, and the scan rate is 10 mV s^−1^. Inset: Tafel plots for the Au-oct-Pt–Ni/C with different Au contents.

Fig. 6 shows the cyclic voltammograms (CVs) recorded in an Ar-saturated electrolyte. All catalysts exhibit characteristic hydrogen adsorption/desorption peaks between 0.05 and 0.4 V, as well as Pt oxidation/reduction peaks between 0.7 and 1.05 V. The intensity of these peaks decreases with increasing Au content, indicating that the Pt surface is partially covered with electrochemically inert Au in the potential region between 0.05 and 1.05 V. At higher potential, however, Au undergoes oxidation, and the corresponding Au oxide reduction charge increases proportionally with the fraction of ECSA loss (Fig. S4). This proportional relationship further supports the interpretation that Au is deposited on the surfaces of the nanoparticles. Fig. 7 shows the linear sweep voltammograms (LSVs) for the oxygen reduction reaction recorded in an O_2_-saturated electrolyte. All catalysts show well-defined diffusion limiting currents in the potential range of 0.3 to 0.7 V. The unmodified 0%Au-oct-Pt–Ni/C, however, exhibits slightly lower currents, and its Tafel plot (inset of Fig. 7) shifts to lower potentials at higher current densities (>1 mA cm^−2^), suggesting higher O_2_-transport resistance, possibly due to less uniform catalyst-layer structure.^29^ Therefore, the mass activity (MA) and specific activity (SA) were evaluated at 0.96 V, where mass-transport effects are negligible.

**Fig. 8:**
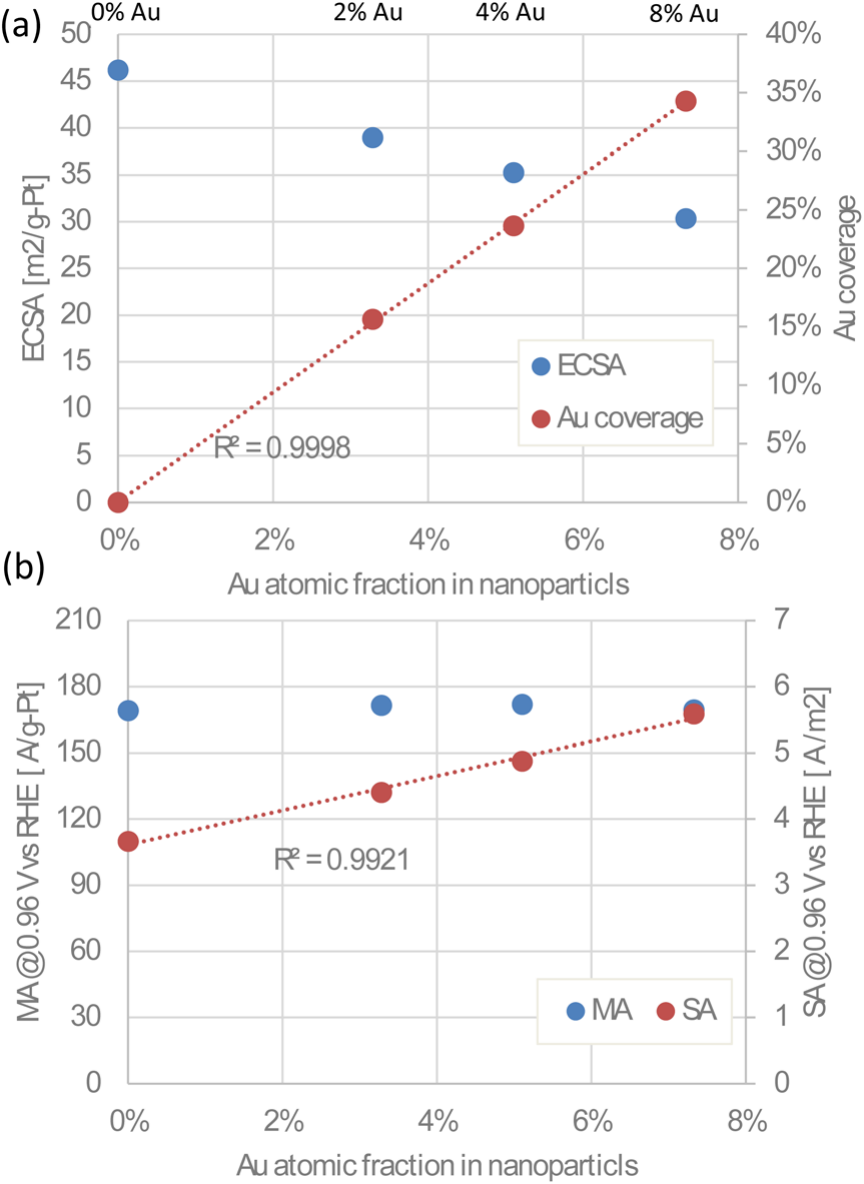
(a) ECSA and Au coverage against the Au atomic fraction in Au-oct-Pt–Ni nanoparticles. (b) MA and SA at 0.96 V against the Au atomic fraction in Au-oct-Pt–Ni nanoparticles.

Fig. 8(a) shows the ECSA and Au coverage plotted against the Au atomic fraction in the Au-oct-Pt–Ni nanoparticles. The Au coverages were defined as the fraction of ECSA loss relative to the Au-unmodified catalyst (0%Au-oct-Pt–Ni/C). The ECSA decreases with increasing Au atomic fraction, while the Au coverage increases linearly. This result confirms that Au was uniformly deposited on the Pt–Ni surfaces at different coverages. If the effects of Au are limited to the Pt–Ni surface adjacent to Au, the Au coverage should be the key parameter determining its influence on ORR activity. Fig. 8(b) shows the MA and SA as a function of the Au atomic fraction in the nanoparticles. The SA increases proportionally with the Au content, reaching approximately 1.5 times higher than that of the unmodified catalyst, whereas the MA remained nearly unchanged owing to the decrease in ECSA. These results are consistent with previous reports, indicating that Au atoms enhance the activity of adjacent Au-free Pt–Ni surface sites through electronic and/or geometric effects.^6^

**Fig. 9:**
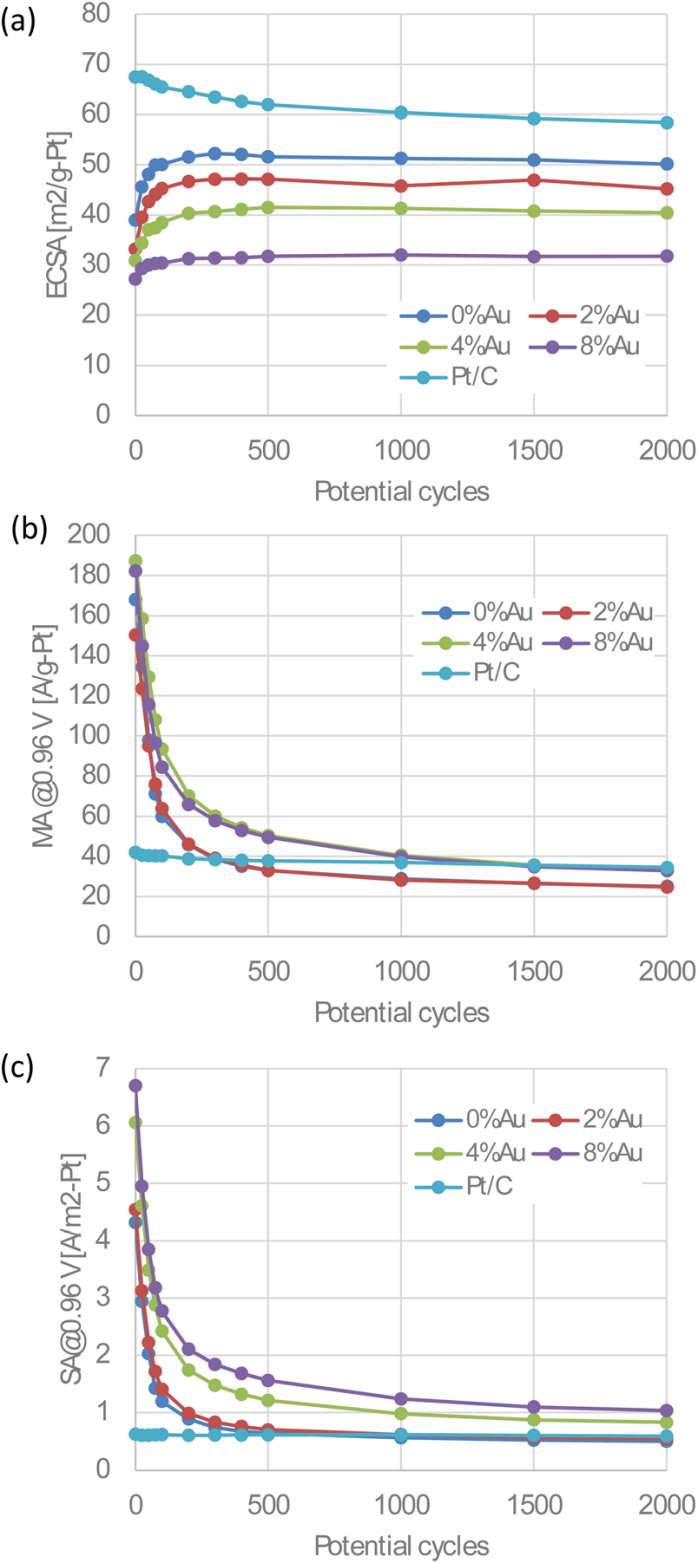
Changes in (a) ECSA, (b) MA, and (c) SA during the accelerated durability test for the Au-oct-Pt–Ni/C with different Au content and Pt/C.

Fig. 9 shows the evolution of the ECSA, MA, and SA during the accelerated durability test (ADT). For reference, the results of a Pt/C catalyst are also presented. All Au-oct-Pt–Ni/C catalysts exhibit a slight increase in ECSA during the initial ∼500 cycles, while both SA and MA decrease sharply over the same period. Our previous report^24^ demonstrated that unmodified oct-Pt–Ni/C undergoes the same degradation, where it arise from the surface roughening of the nanoparticles and a morphological transformation from the octahedral to spherical shape caused by Ni leaching. The present results indicate that the Au-modified catalysts undergo the same structural evolution, demonstrating that the beneficial effect of Au on durability is limited and insufficient to mitigate these degradation processes. Although the degradation rate appears slightly slower at higher Au content, the MAs after the ADT are nearly equal to or lower than that of Pt/C owing to the loss of SA. After the ADT, all Au-modified catalysts exhibit significantly lower SA than their initial values. Nevertheless, catalysts with higher Au contents retain relatively higher SA, whereas those with low Au contents show SA comparable to that of Pt/C. These results indicate that residual Au atoms on the particle surfaces, which may partially dissolve during potential cycling, still enhance the catalytic activity of the adjacent Au-free Pt–Ni surface sites, even though the exceptionally high activities associated with the octahedral shape and optimal Pt–Ni composition are lost. A more detailed understanding of how Au influences degradation, including its dissolution and redistribution during potential cycling, will require further investigation.

The flow-based Au modification approach ensures uniform Au deposition on the nanoparticle surfaces; however, it does not effectively prevent morphological transformation or Ni leaching. Consequently, neither the octahedral shape nor the optimal Pt/Ni composition, which are both crucial for maintaining high ORR activity, are preserved during ADT. The durability enhancement observed here is much less pronounced than that reported for bulk Pt electrodes, spherical nanoparticles, and Pt-based nanowires, where Au modification has been shown to enhance both activity and stability.^9–16^ This difference likely arises from morphology factors: octahedral nanoparticles have sharp edges and corners that are particularly susceptible to dissolution. Therefore, the beneficial effect of Au modification appears to be effective for smooth or well-faceted surfaces but limited for shape-sensitive catalysts with numerous vulnerable edge sites.

## Conclusions

Oct-Pt–Ni nanoparticles (∼6 nm) were synthesized and modified with Au using a continuous flow reactor, and the effects of Au modification on their ORR activity and durability were investigated. Structural analyses confirmed that Pt and Ni form a uniform octahedral alloy nanoparticles and that Au is broadly distributed across the catalyst and partially covers each Pt–Ni nanoparticle. These results demonstrate the effectiveness of the flow-based method for synthesizing morphologically controlled multi-element heterogeneous catalysts, although the specific surface sites covered by Au cannot be identified from the present analysis.

Au modification increased the SA by up to 1.5 times while the MA remained nearly unchanged due to the decrease in ECSA. On the other hand, the ADT showed a rapid decrease in both SA and MA, which is likely caused by Ni leaching and morphological transformation from octahedral to spherical particles.

These results indicate that while Au enhances the intrinsic activity of Pt–Ni surface sites, its ability to suppress Ni dissolution and to preserve the octahedral morphology is limited. Therefore, the durability enhancement effect of Au appears to be restricted for shape-sensitive catalysts such as oct-Pt–Ni nanoparticles.

## Author contributions

T. Nagai conceived and designed the study, performed the experiments, and analysed the data. A. Kuwaki assisted with STEM observation. K. Kodama supervised the project and contributed to data interpretation. All authors discussed the results and contributed to writing the manuscript.

## Conflicts of interest

There are no conflicts to declare.

## Supplementary Material

NA-OLF-D5NA01009H-s001

## Data Availability

The datasets generated and analysed during the current study are available from the corresponding author upon reasonable request. Due to internal data management policies, unrestricted sharing of the raw data may not be possible. Supplementary information (SI) is available. See DOI: https://doi.org/10.1039/d5na01009h.
