# Supplementary material for: Flow-reactor synthesis of octahedral Pt–Ni nanoparticles modified with Au and their evaluation as oxygen reduction reaction catalysts
Source: Nanoscale Adv. 2025 Dec 2;8(2):588–94. doi: 10.1039/d5na01009h (PMC12683288; doi:10.1039/d5na01009h)
Supplement: NA-008-D5NA01009H-s001 [file NA-008-D5NA01009H-s001.pdf]

Supplementary Information (SI) for Nanoscale Advances.
This journal is © The Royal Society of Chemistry 2025

Supplementary information

Flow-Reactor Synthesis of Octahedral Pt-Ni Nanoparticles Modified with Au and Their Evaluation as Oxygen Reduction Reaction Catalysts

Tomoyuki Nagai,*[a] Akira Kuwaki[a] and Kensaku Kodama[a]

[a] *Toyota Central R&D Labs., Inc., 41-1 Yokomichi, Nagakute, Aichi 480-1192, Japan.*

Internal addition method to determine Pt concentrations

The Pt concentration in the sample solution prepared from catalysts was determined by absorption spectroscopy using the internal standard addition method, with $SnCl_2$ as a sensitizer. Tin(II) chloride dihydrate (Super Special Grade), hydrochloric acid (Super Special Grade), and a platinum standard solution (1000 mg/L) were obtained from FUJIFILM Wako Pure Chemical Corporation.

A quartz cuvette (10 mm path length) was filled with 2.7 mL of 0.6 M SnCl2 + 2 M HCl solution. Subsequently, 0.1 mL of the sample solution was added. The mixture was stirred with a small PTFE stirring bar at 1500 rpm for approximately 10s, and then the absorbance at 403 nm was measured using a spectrophotometer (AstraGene II, Astranet Systems Ltd). After the initial measurement, 10 µL of the Pt standard solution was added to the same cuvette, and the absorbance was measurement again. This procedure was repeated four times with increasing additions of the Pt standard solution. Figure S1 shows (a) the absorption spectra for different amounts of the Pt standard solution, and (b) the absorbance at 403 nm plotted as a function of the added Pt standard solution. The x-intercept of the regression line gives the equivalent amount of the Pt standard solution corresponding to the Pt content in 0.1 mL of sample solution. This value was used to calculate the Pt amount in the entire sample solution.

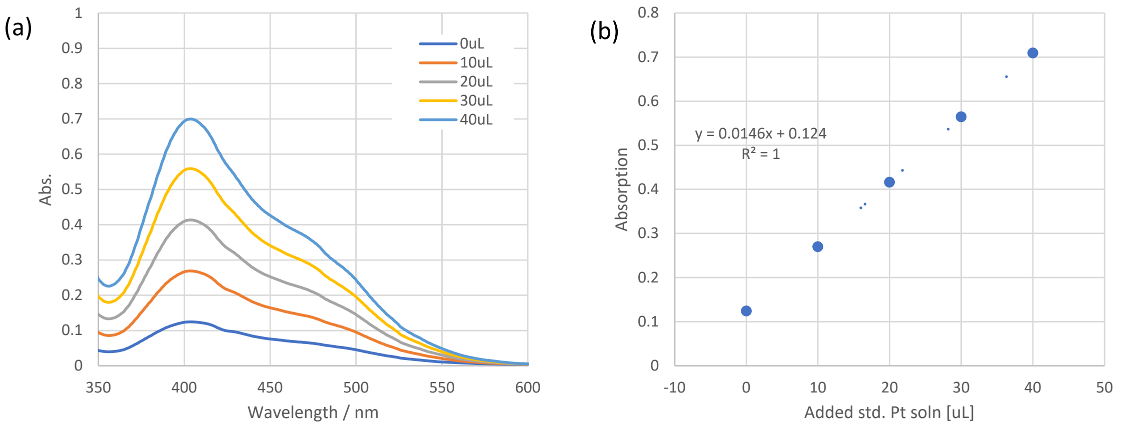

Figure S1 (a) absorption spectra with different amounts of Pt standard solution. (b) absorbance at 403 nm against the amount of Pt standard solution.

Au modification of Pt/C using the conventional two-step Cu-UPD method

For comparison with the continuous-flow synthesis presented in the main text, Au modification of Pt/C with a size of approximately 3 nm in diameter was carried out using the conventional two-step process based on Cu-UPD followed by galvanic replacement with Au. The procedure is briefly summarized below.

1. Loading Pt/C on GC:

Commercial Pt/C catalyst ink was applied onto a glassy carbon (GC) disk and dried to prepare the working electrode.

2. Cu-UPD:

Cu was electrochemically deposited onto Pt/C by immersing the electrode in an electrolyte containing 50 mM $CuSO_4$ and 0.1 M H_2SO_4 and holding the potential at 0.34 V vs. RHE for 3 min, where Cu-UPD occurs and cover the entire Pt surface.

3. Galvanic replacement with Au:

The Cu-modified Pt/C electrode was then immersed in an aqueous solution of 0.01 mM $HAuCl_4$ + 0.1 M $HClO_4$ to induce galvanic replacement between Cu and Au.

Figure S2 shows SEM images of Pt/C on the GC disk before and after Au modification. The conventional Cu-UPD-based 2-step method leads to the formation of aggregated Au nanoparticles (~10 nm) on the top of the Pt/C catalyst layer. The galvanic replacement proceeds very rapidly owing to the large potential difference between Cu dissolution and Au deposition, and can also occur at remote sites through electronic conduction within catalyst aggregates. As a result, Au tends to deposit locally at easily accessible surface regions of these aggregates as shown in Figure S2(c). Because this mechanism is universal to all electronically conductive materials, similar aggregation is expected to occur for octahedral Pt–Ni nanoparticles as well.

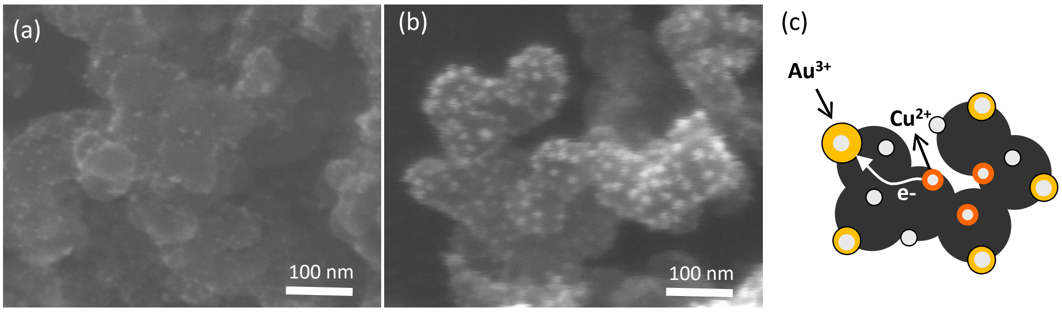

Figure S2 SEM images of (a) Pt/C and (b) Au-Pt/C on the GC disk. (c) The illustrations of galvanic replacement between Cu and Au.

Particle-size distribution of Au-oct-Pt-Ni nanoparticles

Figure S3 shows the particle-size distributions of Au-oct-Pt-Ni nanoparticles with different Au contents. These distributions were obtained by counting approximately 200 particles from STEM images using AIPAS (BLUE TAG Co., Ltd), which is an AI-based analysis software for SEM & TEM particle images. Although the histograms exhibit relatively large deviation due to the limited number of particles, the results indicate that the average particle size remains essentially unchanged upon Au modification.

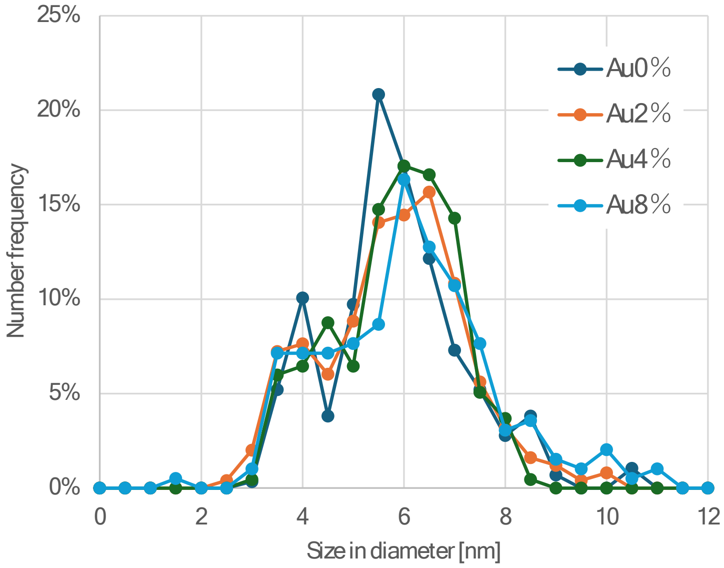

Figure S3 Particle size distribution determined from STEM images of Au-oct-Pt-Ni nanoparticles with different Au contents

High upper-potential cyclic voltammograms for Au-oct-Pt-Ni/C catalysts

Figure S4 shows high upper-potential cyclic voltammograms of the Au-oct-Pt-Ni/C catalysts with different Au contents, recorded in an Ar-saturated 0.1 M $HClO_4$ electrolyte. Clear cathodic peaks corresponding to the reduction of Au oxide are observed around 1.15 V, whereas the anodic Au oxidation peaks are difficult to distinguish owing to overlapping with other reactions such as Pt oxidation, carbon oxidation, and oxygen evolution. Importantly, the Au oxide reduction charge increases approximately proportionally with the fraction of the ECSA loss, namely Au coverage, demonstrating that Au is deposited on the surface of the Pt–Ni nanoparticles.

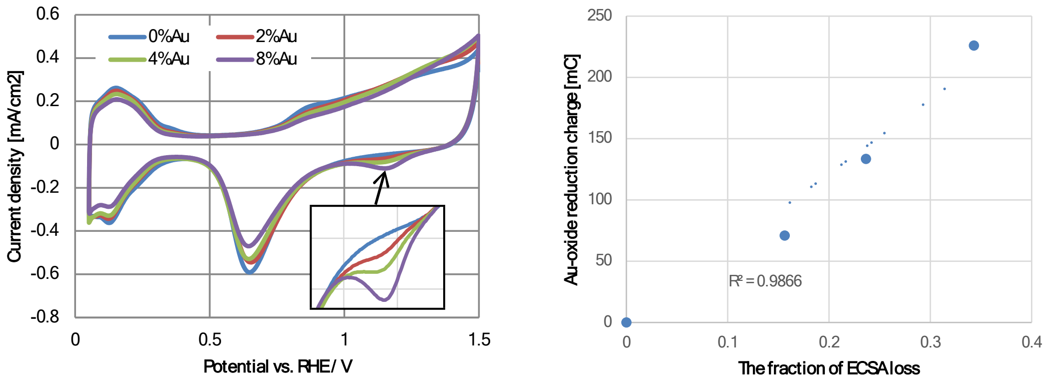

Figure S4 (a) Cyclic voltammograms of the Au-oct-Pt-Ni/C with different Au contents in 0.1 M $HClO_4$ (scan rate: 50 mV/s). (b) Au oxide reduction charge plotted as a function of Au coverage.
